# Editorial: Diagnosis and treatment of gynecologic malignancies during pregnancy

**DOI:** 10.3389/fonc.2023.1143610

**Published:** 2023-01-25

**Authors:** Yudong Wang, Yaru Sheng

**Affiliations:** ^1^ Department of Gynecologic Oncology, International Peace Maternity and Child Health Hospital, School of Medicine, Shanghai Jiao Tong University, Shanghai, China; ^2^ Shanghai Municipal Key Clinical Specialty, Female Tumor Reproductive Specialty, Shanghai, China; ^3^ Shanghai Key Laboratory of Embryo Original Diseases, Shanghai Jiao Tong University, Shanghai, China

**Keywords:** gynecologic oncology, pregnancy, chemotherapy, surgery, fertility

Gynecologic oncofertility involves the diagnosis and treatment of gynecological tumors during pregnancy and fertility protection in gynecologic oncology patients. The diagnosis and treatment of gynecological malignancies during pregnancy is a great challenge because both tumor management and maternal-fetal safety should be taken into consideration as some tumor markers can be affected by physiological or pathological variations during pregnancy. Therefore, its role in the diagnosis and treatment of ovarian tumors in pregnancy is limited, and the continuous enlargement of the uterus and the physiological changes during pregnancy make diagnosis and treatment more difficult.

Cervical cancers in pregnancy are mostly diagnosed at early stages and have favorable outcomes (1); however, stage IB3 tumors remain a challenge for physicians (2). In addition, pregnant patients with cervical lesions show a relatively high proportion of combined high-risk human papillomavirus infections (3). There is evidence that combined PCDHGB7 methylation detection may help in screening for cervical tumors during pregnancy (4). Malignant ovarian tumors were reported in 0.85 per 10,000 deliveries, ranging from 0.44 to 1.71 per 10,000 pregnancies (5), with mature cystic teratoma being the most common benign ovarian tumor in patients with adnexal masses; this was an independent risk factor for premature rupture of membranes (6). We further explored the risk factors for gynecological tumors and the clinical prognostic benefits of adjuvant chemotherapy and investigated the impact of malignant tumors on the outcomes of assisted reproductive pregnancy.

For patients with gynecological cancer, the beneficial effect of metformin use remains controversial. A meta-analysis conducted by Yao et al. showed no significant association between metformin use and the risk of gynecologic cancer in diabetes mellitus. In addition, no significant association was observed between metformin use and recurrence-free survival and cancer-specific survival among patients with gynecologic cancer and diabetes mellitus, which indicated that metformin may be a useful adjuvant agent for gynecological cancer with diabetes mellitus because it does not increase the risk of gynecologic cancer, especially for patients with ovarian and endometrial cancers.

Studies on whether a history of cancer affects long-term reproductive outcomes in women who undergo assisted reproductive technology (ART) are scarce. Here, Li et al. performed a retrospective case-control study, which demonstrated that women with a history of cancer can conceive using ART and that their long-term reproductive outcomes are similar to these of healthy infertile women. A history of cancer does not decrease the number of retrieved oocytes, increase the risk of adverse obstetric outcomes, or affect birth outcomes.

Long et al. screened patients with stage I mucinous ovarian cancer (MOC) from the Surveillance, Epidemiology, and End Results database and found that patients aged 31–45 years, with grade 3, stage IC, who underwent non-fertility-sparing surgery were more likely to receive adjuvant chemotherapy. For stage I patients with MOC who underwent fertility-sparing surgery, adjuvant chemotherapy may increase the risk of death; reduced cancer-specific survival was also observed in chemotherapy groups in patients with stage IA/IB-grade 2 MOC.

Daley et al. reported a unique case: a 28-year-old woman was diagnosed with early-stage grade 1 endometrioid endometrial cancer and ovarian cancer with histopathology of well-differentiated endometrioid carcinoma. In light of her young age and nulliparity, she was offered hormonal treatment with oral megestrol acetate 160 mg daily. However, she stopped taking megestrol acetate after 2 weeks because of intolerable side effects and did not undergo surgery. However, the endometrial and ovarian cancers showed completely spontaneous regression following pregnancy in the absence of any comprehensive intervention. Postpartum follow-up ultrasound showed that the appearance of the uterus and ovaries and tumor marker levels were normal. Hysteroscopy revealed normal endometrium with no polyps and no evidence of hyperplasia or malignancy. At 8 months postnatally, the patient’s menstrual periods recommenced, with no menstrual irregularities or intermenstrual bleeding.

This unique case demonstrates the potential for pregnancy to induce remission of gynecological malignancies, which may be related to the high progesterone-producing state of pregnancy. The modification of current progestin therapy regimens to achieve pregnancy levels of progesterone may further improve the prognostic benefit of fertility-sparing treatment options for low-grade endometrial cancers. Such benefits may also be applicable to borderline or early-stage ovarian cancers in the future.

In general, these studies increase our understanding of gynecological malignancies during pregnancy, provide new inspiration for expectant treatment or surgery, and improve the prognosis of patients.

## Author contributions

YS wrote the paper and YW contributed to review and edit the editorial for this Research Topic. All authors contributed to the article and approved the submitted version
